# Modern Epidemics: From the Spanish Flu to COVID-19

**DOI:** 10.3201/eid2712.211312

**Published:** 2021-12

**Authors:** Megan A. Greischar

**Affiliations:** Cornell University, Ithaca, New York, USA

**Keywords:** epidemics, severe acute respiratory syndrome coronavirus 2, SARS-CoV-2, SARS, coronaviruses coronavirus disease, COVID-19, zoonoses, viruses, influenza, respiratory diseases

In Modern Epidemics: From the Spanish Flu to COVID-19, Salvador Macip presents an ambitiously comprehensive overview of human diseases (Figure). This updated version, translated into English by Julie Wark, provides an accessible introduction to the microbes that cause harm, the science behind treatment and prevention, and the challenges to successful disease control. The book begins, appropriately, with how bacteria have enabled humans to evolve and persist and then pivots to pathogenic microbes.

**Figure:**
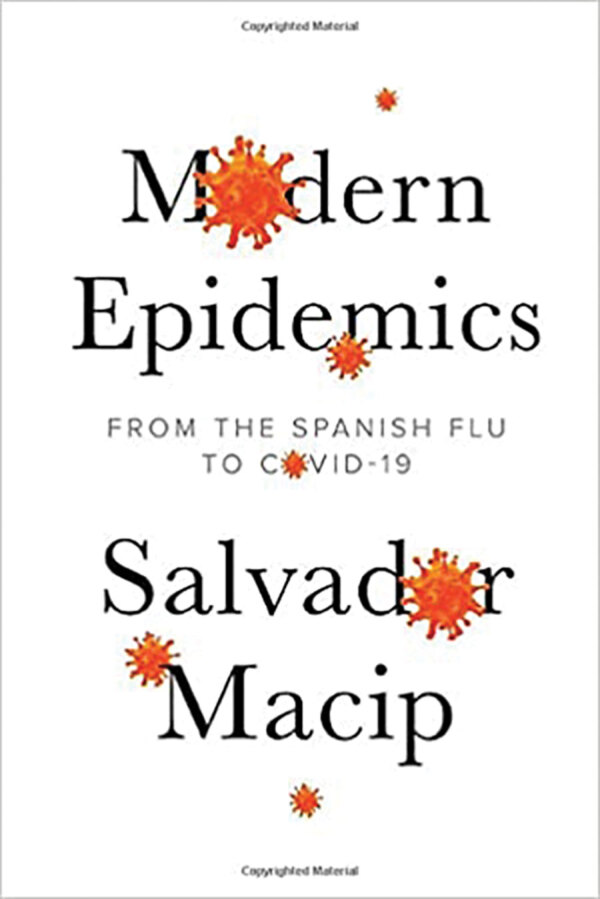
Modern Epidemics: From the Spanish Flu to COVID-19

Modern Epidemics is written with lay readers in mind, so diseases are grouped by relevance to humans—for instance microbes with bioterrorist potential—rather than by pathogen relatedness. Macip guides readers through tools for prevention and treatment, and current knowledge for neglected and emerging diseases, including coronaviruses. 

Unsurprisingly, coronavirus disease (COVID-19) information has become rapidly outdated, including the death toll, but that chapter maintains relevance as a time capsule of the early pandemic. Some uncertainties Macip relates also have been resolved; for instance, the promise of the Moderna vaccine has been borne out. Still, some uncertainties remain, such as whether COVID-19 will persist as a seasonal disease, like influenza or a mild cold-like illness, or as isolated outbreaks, damaging but limited in scope. 

Macip describes the impossibility of predicting future pandemics, which could emerge from diverse microbes or, as he states, “a supervirus that doesn’t even exist yet,” but I suspect such predictions could become possible. Understanding why pathogens evolve to cause harm represents a major focus in evolutionary biology. Indeed, recent research investigates why pathogens evolve transmission before symptoms (*1*), a trait responsible for considerable COVID-19 spread. This perspective narrows the possibilities by examining whether harmful traits are unlikely to emerge or simply have not evolved yet (*2*). Macip instead addresses the more immediate question of how to plan for the worst and incorporate data as it becomes available.

Modern Epidemics ends with an overview of major ongoing epidemics, including influenza, HIV, tuberculosis, and malaria. Macip explains the mechanics of producing influenza vaccines, including why producing vaccines against certain strains can be difficult; the surprising reason is that some viruses replicate agonizingly slowly inside chicken eggs. Despite impressive scientific gains, these epidemics continue to impose an enormous health burden; discussing them last underscores the substantial challenges that remain even after the COVID-19 pandemic.

Throughout Modern Epidemics, Macip avoids idolizing preeminent scientists, past and present. For example, he points out that immunization had precedent in other cultures long before it was supposedly discovered by Edward Jenner. Crucially, Macip’s narrative continues beyond the scientific discovery of effective treatments or preventative measures to outline the enormous political and economic barriers that persist despite scientific advances. Unfortunately, the comprehensiveness of Modern Epidemics precludes in-depth exploration of fascinating topics that arise during these narratives, such as the use of disease as a weapon of colonization and the economics of sustaining disease control. Nonetheless, Modern Epidemics serves as a broad-ranging introduction to the history, biology, and sociology of infectious diseases and will be useful to readers wishing to rapidly gain a sense of the field.
